# Hyperglycemia Induces the Variations of 11**β**-Hydroxysteroid Dehydrogenase Type 1 and Peroxisome Proliferator-Activated Receptor-**γ** Expression in Hippocampus and Hypothalamus of Diabetic Rats

**DOI:** 10.1155/2012/107130

**Published:** 2012-06-26

**Authors:** Wen-wen Qi, Li-yong Zhong, Xiao-rong Li, Guang Li, Zhao-xia Liu, Jin-feng Hu, Nai-hong Chen

**Affiliations:** ^1^Department of Endocrinology, Department of Pathology, Beijing Tiantan Hospital, Capital Medical University, No. 6 Tiantan Xili Road, Dongcheng District, Beijing 100050, China; ^2^Institute of Materia Medica, Peking Union Medical College, Chinese Academy of Medical Sciences, No. 1 Xian Nong Tan Street, Xicheng District, Beijing 100050, China

## Abstract

In this paper, we first observed that there were differences in expressions of 11**β**-HSD1 and PPAR-**γ**, in hippocampi and hypothalami, among constant hyperglycemia group, control group and the fluctuant glycemia group, using Immunohistochemical analysis. However, whether in expression o f 11**β**-HSD1 or PPAR-**γ**, there were no statistic differences between the control group or the fluctuant glycemia group. So, we removed the fluctuant glycemia group, retaining only constant hyperglycemia group and control group, being fed for 8 weeks. After 8 weeks of induction, 11**β**-HSD1 expression increased and PPAR-**γ** expression decreased in the constant hyperglycemia group compared with control group, both in hippocampi and hypothalami, by Western Blot. The constant hyperglycemia group also showed impaired cognition in MORRIS watermaze, lower serum corticosterone level, and higher Serum ACTH concentration after 8 weeks. We inferred that the cognition impairment may be related to the abnormal expression of 11**β**-HSD1 and PPAR-**γ** in central nerves system. As for 11**β**-HSD1 is a regulating enzyme, converting the inactive 11-dehydrocorticosterone into the active glucocorticoid corticosterone, thus amplifying GC action in local tissues. It is also well known that high local GC levels can affect the cognitive function. In addition, PPAR-a protective receptor, which is related to cognition.

## 1. Introduction

Diabetes mellitus negatively affects the cognitive function, neurophysiology, and structure in the brain, which is referred as diabetic encephalopathy. Diabetic encephalopathy is associated with an increased risk of Alzheimer's disease (AD) and dementia [1]. Some studies have demonstrated that hyperglycemia, hyperlipidemia, the change of blood flow, and neurotrophic factors in diabetes [2–5] are involved in the pathophysiology of diabetic encephalopathy. The mechanism of diabetes on cognitive impairment, however, remains unidentified. Therefore, in this study we investigated the influence of hyperglycemia on cognition and its mechanism.

11*β*-Hydroxysteroid dehydrogenase type 1 (11*β*-HSD1) converts inactive 11-dehydrocorticosterone into active glucocorticoid corticosterone (GC) in vivo, playing an important role in regulating the level of the glucocorticoid, with a general distribution in liver, adipose tissue, cerebral cortex, hippocampus, amygdale, and so forth [6, 7]. The peroxisome proliferator-activated receptors (PPARs) are the members of the nuclear receptor superfamily, which can regulate gene expression [8]. PPAR-*γ* is an isoform of PPARs, expressed mainly in adipose tissue. It acts on cell differentiation, lipid metabolism, and insulin sensitivity [9]. It has been reported that abnormal elevation of 11*β*-HSD1 activity in brain may contribute to the development of AD, which is characterized by cognitive impairment [10]. Furthermore, AD is related to the decreased levels of PPAR-*γ* in the brain [11]. These studies may suggest a hypothesis that the cognitive impairment in diabetes may be associated with the abnormal levels of 11*β*-HSD1 and PPAR-*γ* in the brain.

Therefore, we hypothesize that hyperglycemia would induce the variations of 11*β*-HSD1 and PPAR-*γ* expressions in hippocampus and hypothalamus, which are related to the cognitive impairment in diabetic rats. In this study, we demonstrated that the variations of 11*β*-HSD1 and PPAR-*γ* expressions in hippocampi and hypothalamic, respectively, in STZ-induced SD rats are detected by immunohistochemistry and western blot. Wealso measured the variations of hormones within HPA axis. Furthermore, the changes of cognitive function of these experimental diabetic rats had been detected by the Morris water maze during the experimental period, before the samples had been collected as well.

## 2. Materials and Methods

### 2.1. Reagents

Streptozotocin (STZ) was purchased from Sigma Chemical Company (USA). Anti-rat 11*β*-HSD1 primary antibody and PPAR-*γ* primary antibody for western blot were from Abcam Biotechnology (England). Anti-rat 11*β*-HSD1 primary antibody for immunohistochemistry was from Abcam Biotechnology (England). Anti-rat PPAR-*γ* primary antibody for immunohistochemistry was from Santa Cruz Biotechnology (USA). Adrenocorticotropic hormone (ACTH) and corticosterone rat ELISA kits were from Rapidbio Biosource Company (USA).

### 2.2. Research Design

Male Sprague-Dawley (SD) rats, weighing 180–250 g, were obtained from the Capital Medical University, the Animal Research Institute of Beijing Stomatological Hospital. Rats were housed at 22 ± 2°C, on a 12-hour light-dark cycle, and fed with standard laboratory chow and water ad libitum. STZ was dissolved in 0.1 mol/L citrate buffer solution (PH 4.2–4.5) before injection. The animals were injected by streptozotocin at the dose of 70 mg/kg of the body weight intraperitoneally to induce diabetes. Rats with tail blood glucose levels ≥16.7 mM in 3 days were considered diabetic. In protocol 1, rats were distributed into 3 groups and raised for 2 weeks: (1) constant hyperglycemia group (*n* = 10) were allowed free access to water and standard chow diet; (2) fluctuant glycemia group (*n* = 10) received regular insulin (3 u/per day once a day at 10 : 00) subcutaneously and were isolated from any food intake for 2 hours; (3) control group (*n* = 10) received an equal volume of citrate buffer solution. In protocol 2, rats were divided into constant hyperglycemia group (*n* = 13) and control group (*n* = 9) and raised for 8 weeks. After 8 weeks 4 rats died in the constant hyperglycemia group, so that every group has 9 rats. The 11*β*-HSD1 and PPAR-*γ* expressions in hippocampus and hypothalamus were detected by immunohistochemistry in protocol 1 and western blot in protocol 2. Serum ACTH and corticosterone concentrations were detected by ELISA. The change of cognitive function in diabetic rats raised 8 weeks after induction was detected by Morris water maze. This study was approved by the animal care and use committee of the Capital Medical University.

### 2.3. MORRIS Water Maze

The Morris water maze is a circular water tank (200 cm in diameter and 50 cm in height), filled with water maintained at 22–24°C at the height of 30 cm. A platform was set 2 cm below the water inside the tank. The whole tank was divided into 4 quadrants. Two trails were performed as follows: (1) hidden platform trial: each rat was put into the water from four starting positions, the sequence of which was fixed. A rat was trained for 5 consecutive days. During the test trial, a rat was placed into the tank at the same starting point, with his head facing to the wall. The time from putting into the water to reaching the platform was measured. If failed to climb onto the platform within 60 seconds, the rat would be guided onto the platform and the time was recorded as 60 seconds. After finishing a trail, the rat stayed on the platform for 15 seconds before starting next trial; (2) probe trial: on the sixth day, the platform was removed. The swimming routes within one minute and the times crossing the platform area were recorded.

### 2.4. Immunohistochemical Analysis

A rat was perfused with 10% paraformaldehyde in 0.1 mol/L phosphate buffer (pH 7.4). Hippocampi and hypothalami were separated from the whole brain tissue. Then they were dehydrated, cleaned, paraffin imbedded, and cut into 5 *μ*m thick slices. After slices were incubated with 11*β*-HSD1 antibody, PPAR-*γ* antibody, or PBS (negative control) at 4°C overnight, they were covered with secondary antibody for 20 minutes at 37°C. Finally they were stained with DAB/H_2_O_2_. The gray values of 11*β*-HSD1 and PPAR-*γ* immunoreactants in the hippocampus and hypothalamus were analyzed using Image-Pro Plus Version 6.0 color image analysis system.

### 2.5. Western Blot

The homogenate of hippocampi or hypothalami was centrifuged at 15,000 rpm for 20 minutes at 4°C, and the supernatants were obtained for western blot. Briefly, samples were separated by SDS-polyacrylamide gel electrophoresis. Proteins were transferred to PVDF membranes, and the membranes were blocked with 3% BSA Tris- buffer saline and probed with primary antibodies against 11*β*-HSD1 (1^ ^: 1000), PPAR-*γ* (1 : 1000), *β*-actin (1 : 1000), or Biotin-conjugated anti-rat IgG (1 : 5000) for 11*β*-HSD1 for 2 h at room temperature. This was followed by a secondary antibody coupled to horseradish peroxidase, and the blots were developed using enzyme-linked chemiluminescence. Autoradiographic films were scanned densitometrically and quantitated by densitometric analysis. 

### 2.6. Measurement of Serum ACTH and Corticosterone

Animals were anesthetized with an intraperitoneal injection of 1.5% phenobarbital (30 mg/kg) and fixed in supine position. 5 ml blood was drawn directly from the heart after rat chest was opened. Then the blood was storeod at room temperature for 3 hours and centrifuged at 3000* g for 10 min. The serum was collected and stored at −80°C. ACTH and corticosterone concentrations were measured by ELISA, according to the manufacturer's protocols.

### 2.7. Statistical Analysis

All the data, standardized into Z-scores, were presented as mean ± SD and analyzed with one-way ANOVA. The data, unstandardized into Z-scores, were presented as the median (interquartile range) and analyzed with the Wilcoxon rank sum test. The result with *P*  value < 0.05 was considered statistically significant. Statistical analyses were performed with SPSS16.0 software.

## 3. Results 

### 3.1. Characteristics of the Rats

**Figure 1:**
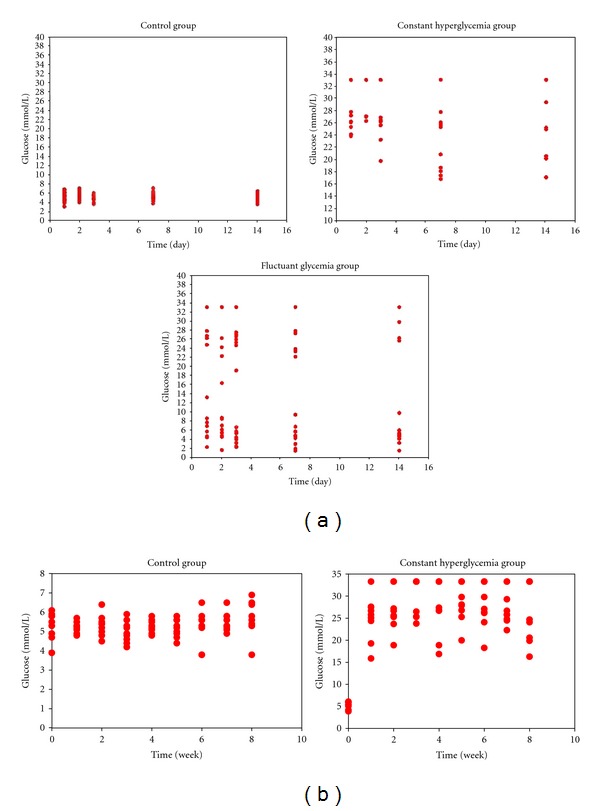
(a) Glycemia profile in control group, fluctuant glycemia group, and constant hyperglycemia group from 0 to 14 days after induction. (b) Glycemia profile in control group and in constant hyperglycemia group from 0 to 8 weeks after induction.

After 2 weeks of induction, constant hyperglycemia group had sustained hyperglycemia, and fluctuant glycemia group had large fluctuations in blood glucose, with control group having normal blood glucose (Figure 1(a)). So, the models were induced successfully. After 8 weeks of induction, 4 rats in the constant hyperglycemia group died, while all the rats in the control group were alive group. The constant hyperglycemia group had sustained hyperglycemia, with control group having normal blood glucose (Figure 1(b)).

### 3.2. 11*β*-HSD1 Expression in Hippocampus and Hypothalamus

**Figure 2:**
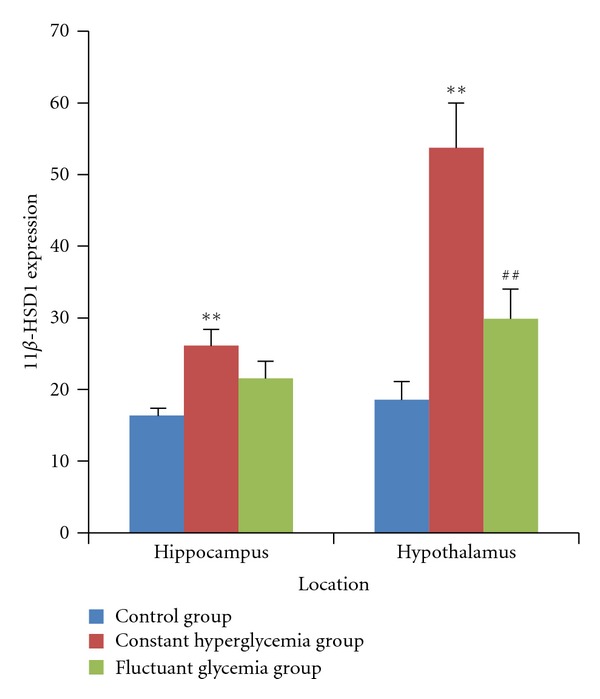
11*β*-HSD1 expression in hippocampus and hypothalamus measured by immunohistochemical techonology after 2 weeks of induction. In hippocampi, 11*β*-HSD1 expression were increased in the constant hyperglycemia group (*n* = 10) compared with that in the control group (*n* = 10) (*P* < 0.05). In hypothalami, 11*β*-HSD1 expression were increased in the constant hyperglycemia group (*n* = 10) compared with that in the control group (*n* = 10) (*P* < 0.05) and with the fluctuant glycemia group (*n* = 10) (*P* < 0.05). **P < 0.01, versus control group. ^##^ *P* < 0.01, versus constant hyperglycemia group.

**Figure 3:**
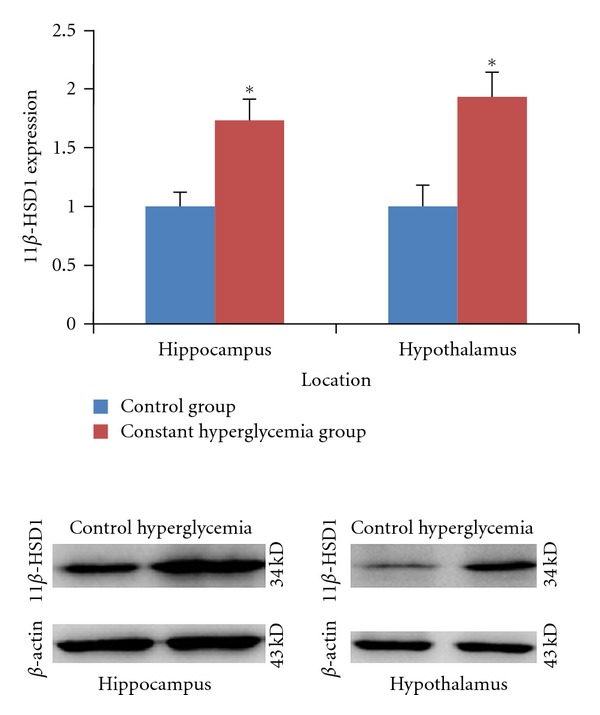
11*β*-HSD1 expression in hippocampus and hypothalamus measured by western blot after 8 weeks of induction. 11*β*-HSD1 expression was increased in the constant hyperglycemia group (*n* = 9) compared with that in the control group (*n* = 9) in hippocampi (*P* < 0.05) and hypothalami (*P* < 0.05). *P < 0.05, versus control group.

**Figure 4:**
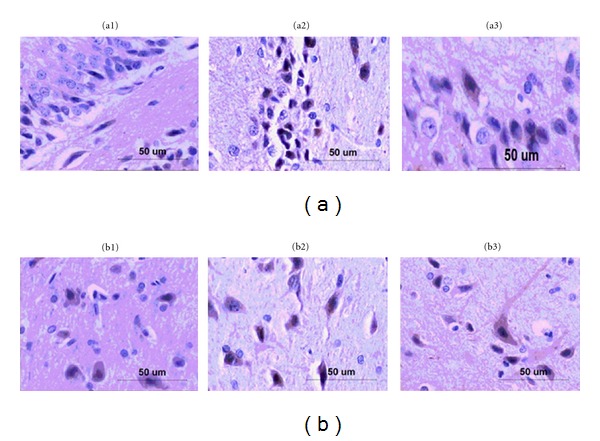
11*β*-HSD1 expression in hippocampus and hypothalamus by immunohistochemical techonology. (a) 11*β*-HSD1 expression in hippocampus (by light microscopy × 400) (a1): control group (*n* = 10), (a2): constant hyperglycemia group (*n* = 10), (a3): fluctuant glycemia group (*n* = 10). (b) 11*β*-HSD1 expression in hypothalamus (by light microscopy × 400). (b1): control group (*n* = 10), (b2): constant hyperglycemia group (*n* = 10), (b3): fluctuant glycemia group (*n* = 10). There were positive staining of 11*β*-HSD1 (yellow-brown particles) in all groups. In hippocampus and hypothalamus, the constant hyperglycemia group had the most 11*β*-HSD1-positive expressions and the control group had the least 11*β*-HSD1-positive expressions.

We firstly demonstrated that 11*β*-HSD1 was present in both hippocampi and hypothalami using immunohistochemical techonology. In Figure 2 and Figure 4, after 2 weeks of induction, compared with the control group, 11*β*-HSD1 expression increased in hippocampi in the constant hyperglycemia group (*P* < 0.05). In hypothalami, 11*β*-HSD1 expression increased in the constant hyperglycemia group compared with the control group (*P* < 0.05) or the fluctuant glycemia group (*P* < 0.05). In Figure 3, The constant hyperglycemia rats of 8 weeks of induction also showed a markedly higher level of 11*β*-HSD1 in both hippocampi and hypothalami compared with the control rats (*P* < 0.05). 

### 3.3. PPAR-*γ* Expression in Hippocampus and Hypothalamus

**Figure 5:**
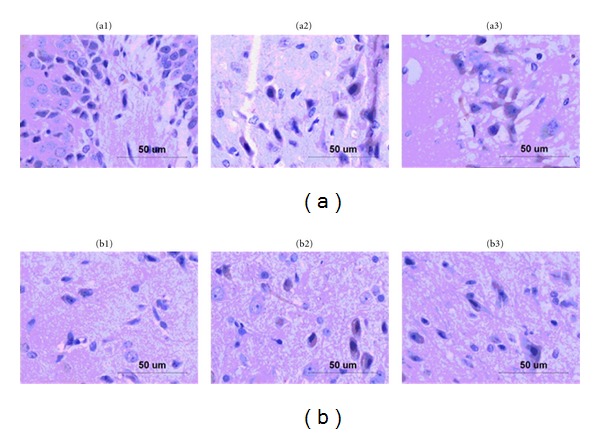
PPAR-*γ* expression in hippocampus and hypothalamus by immunohistochemical technology. (a) PPAR-*γ* expression in hippocampus (by light microscopy × 400). (a1): control group (*n* = 10), (a2): constant hyperglycemia group (*n* = 10), (a3): fluctuant glycemia group (*n* = 10). (b) PPAR-*γ* expression in hypothalamus (by light microscopy × 4000). (b1): control group (*n* = 10), (b2): constant hyperglycemia group (*n* = 10), (b3): fluctuant glycemia group (*n* = 10). There were positive stainings of PPAR-*γ* (yellow-brown particles) in all groups. In hippocampus and hypothalamus, the constant hyperglycemia group had the most PPAR-*γ*-positive expressions and the control group had the least PPAR-*γ*-positive expressions.

**Figure 6:**
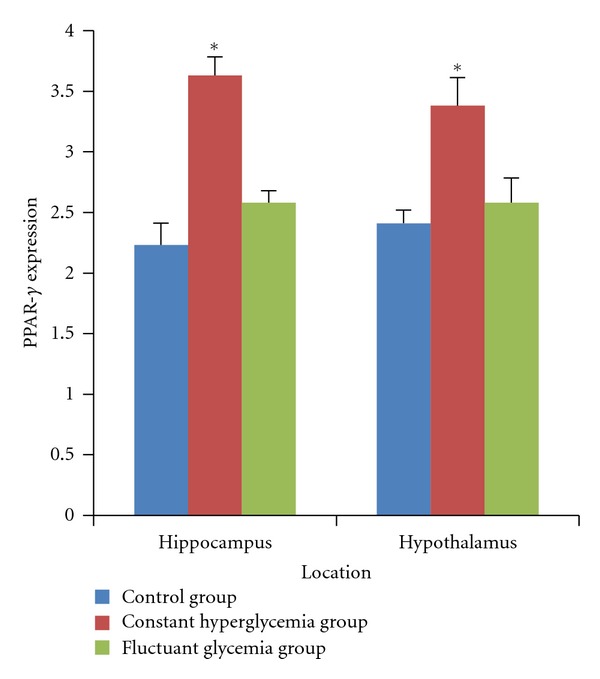
PPAR-*γ* expression in hippocampus and hypothalamus measured by immunohistochemical technology after 2 weeks of induction. In hippocampi and hypothalami, PPAR-*γ* expression increased in the constant hyperglycemia group (*n* = 10), compared with the control group (*n* = 10; *P* < 0.05). *P < 0.05, versus control group.

**Figure 7:**
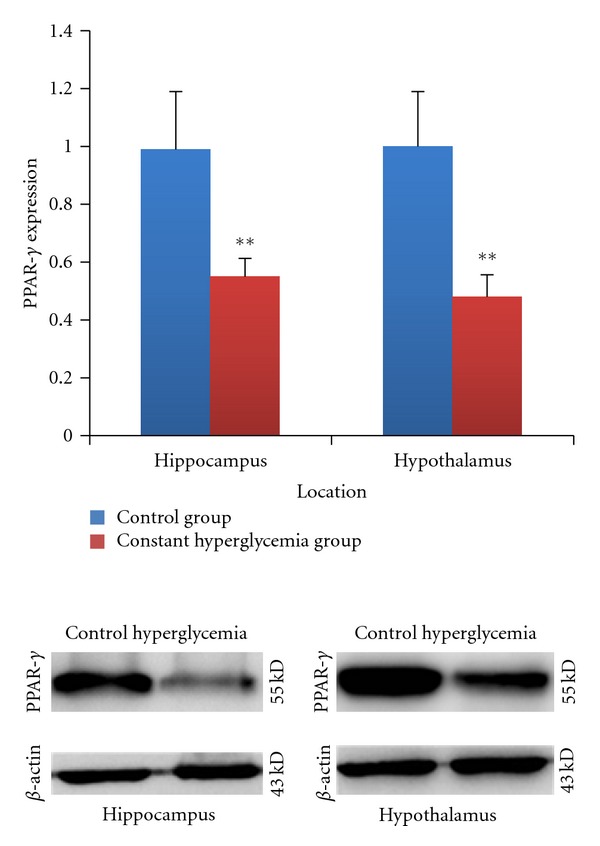
PPAR-*γ* expression in hippocampus and hypothalamus measured by western blot after 8 weeks of induction. PPAR-*γ* expression decreased in the constant hyperglycemia group (*n* = 9) compared with the control group (*n* = 9) in hippocampi (*P* < 0.01) and hypothalami (*P* < 0.01). **P < 0.01, versus control group.

Then we examined PPAR-*γ* expression in hippocampi and hypothalami by immunohistochemical and western blot, respectively. In Figure 5, PPAR-*γ* was expressed in both hippocampi and hypothalami. In Figure 6, after 2 weeks of induction, PPAR-*γ* expression in hippocampi was significantly higher than that in the control group (*P* < 0.05). In Figure 7, After 8 weeks of diabetes, we found that PPAR-*γ* expression was decreased in the constant hyperglycemia group both in hippocampi and hypothalami (*P* < 0.01) compared with the control group assayed by western blot.

### 3.4. Spatial Learning and Memory

**Figure 8:**
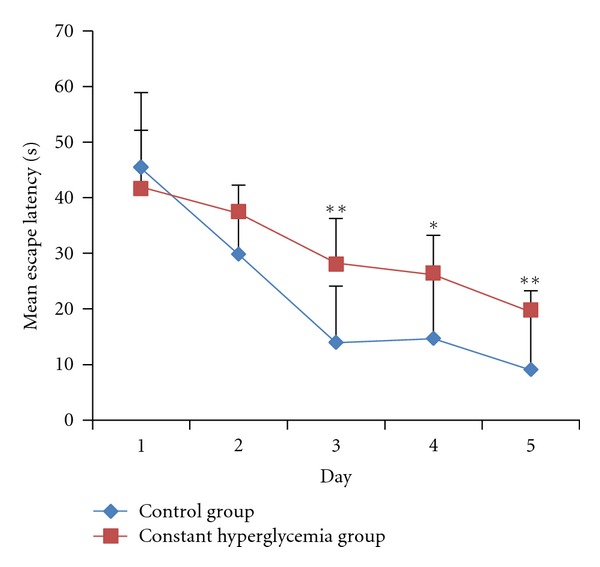
Mean escape latencies in the hidden platform trial. In the first two days, there were no significant differences statistically in the escape latencies between two groups. After two days, the constant hyperglycemia group (*n* = 9) had a longer escape latencies than control group (*n* = 9). **P < 0.01 versus control group, *P < 0.05 versus control group.

**Figure 9:**
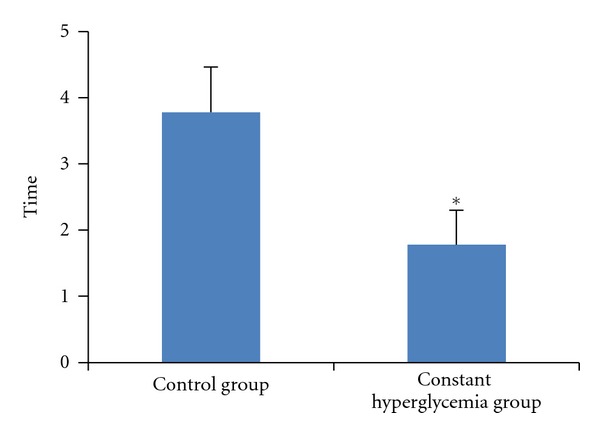
The time of crossing the platform area in the probe trial. The time of crossing the platform area was significantly decreased in the constant hyperglycemia group (*n* = 9) than control group (*n* = 9). *P < 0.05, versus control group.

Next we tested the spatial learning and memory abilities in the constant hyperglycemia rats and the control rats after 8 weeks of induction. In Figure 8, in the hidden platform trial, the time to locate the platform decreased by a time-dependent manner in consecutive 5 days within the two groups. Although there was no significant difference in the escape latencies between the two groups at the first two days, the escape latencies in the constant hyperglycemia group were longer than that in the control group starting from the third day (*P* < 0.01). In Figure 9, in the probe trial, the time of crossing the platform area was significantly decreased in the constant hyperglycemia group (*P* < 0.05). These results suggest that the rats with constant hyperglycemia had cognitive impairment, compared with the healthy rats. 

### 3.5. Serum Levels of ACTH and Corticosterone

In two-week induction experiment, serum ACTH level in the constant hyperglycemia group was higher than that in the control group (*P* < 0.01) and the fluctuant glycemia group (*P* < 0.01). Serum ACTH levels of the fluctuant glycemia group were higher than the control group (*P* < 0.01). By the time of eight weeks after induction, the constant hyperglycemia group had lower serum corticosterone levels (*P* < 0.01) and higher serum ACTH levels (*P* < 0.01), compared with the control group (*P* < 0.01, Table 1).

## 4. Discussion

In this study, we observed that 11*β*-HSD1 and PPAR-*γ* expressions in the constant hyperglycemia rats underwent a dramatic increasing in hippocampi and hypothalami, compared with that in the control rats after 2 weeks of induction. However, there was no statistic difference in 11*β*-HSD1 and PPAR-*γ* expression between the control group and the fluctuant glycemia group. We then raised the diabetic rats for 8 weeks for assaying 11*β*-HSD1 and PPAR-*γ* expressions in hippocampi and hypothalami using western blot. After 8 weeks of induction, 11*β*-HSD1 expression in the constant hyperglycemia group increased in both hippocampi and hypothalami. Interestingly, PPAR-*γ* expression of the constant hyperglycemia rats decreased significantly in both hippocampi and hypothalami, compared with the control rats. Therefore, the results suggest that 11*β*-HSD1 expression increased in hippocampi and hypothalami in the condition of hyperglycemia, whereas PPAR-*γ* expression in hippocampi and hypothalami increased during the early stage of hyperglycemia and then decreased. We also observed that the constant hyperglycemia group showed longer escape latencies and less time of crossing the platform area after 8 weeks of induction, implying that the constant hyperglycemia rats had cognitive impairment compared with the control rats. Kamboj SS reported that STZ-induced diabetes produced marked impairment in cognitive function after 8 weeks of induction [1], which is compatible with our result.

11*β*-HSD1 is a regulating enzyme, converting inactive 11-dehydrocorticosterone into active glucocorticoid corticosterone in rodents, thus amplifying GC action in local tissues [6]. Local GC level can affect the cognitive function. The normal level of GC is essential for maintaining the development of the neural cells and the process of studying and memory. However, a higher local level of GC probably contributes to the cognitive impairment and the impairment of the memory form by suppressing the hippocampus BDNF expression, which is a neurotrophic factor [12]. Alzheimer disease (AD), characterized by cognitive impairment, is related to abnormal elevation of 11*β*-HSD1 activities in brain [10]. A number of studies show that aged 11*β*-HSD1 knock-out mice have significant enhancement in LTP and obvious decline in latencies in water maze task compared to the wild aged mice, suggesting that the knockout of 11*β*-HSD1 gene might protect from the documented age-related decline in neurogenesis [6, 13]. It has been reported that adrenalectomized rats with basal GC replacement and additional GC treatments for 3 days showed reduced synaptic strength and increment in long-term depression at medial perforant path synapses and impairment of spatial reference memory [14]. Expression of 11*β*-hydroxysteroid dehydrogenase type II (11*β*-HSD2), mostly in mature dentate gyrus granule cells, can reverse the effects of high GC level [14] while 11*β*-HSD2, an isozyme of 11*β*-HSD1, has the adverse effects on GC, acts as a potent 11*β*-dehydrogenase, and inactivates rapidly glucocorticoids [15]. All these reports imply that blockage or deregulation of the expression of 11*β*-HSD1 results in a neuroprotective profile. Adversely, a higher level of local GC in central nervous system will produce atrophy of dendrites, neuronal and cognitive impairment, and even neuronal loss in some strains of rats [15]. However, there are minor studies about whether cognitive impairment is related to the abnormal expression of 11*β*-HSD1 in central nervous system in diabetes. In the present study, we demonstrated that constant hyperglycemia induced a higher level of 11*β*-HSD1 in hippocampus and hypothalamus in the rats after 2 or 8 weeks of induction. Moreover, the constant hyperglycemia produced an impaired cognition in rats. All these data strongly suggest that the cognitive impairment in diabetes is probably associated with the increased local GC level in central nervous system induced by the increased 11*β*-HSD1.

It is reported that PPAR-*γ* agonists have protective effect within the brain [16]. Also some researchers regard PPAR-*γ* as a protective receptor [17]. It has been proved that insulin-degrading enzyme (IDE), which can reduce the levels of brain A *β* and insulin, is associated with AD and type 2 diabetes mellitus. PPAR-*γ* can transcriptionally induce IDE expression and its level decreases in AD and T2DM subjects, which results in a lower level of IDE in AD and T2DM subjects [11]. The evidence shows that a PPAR-*γ* agonist, pioglitazone, can attenuate the cognitive impairment of rats injected by STZ intracerebroventricularly. These facts imply that the abnormal level of PPAR-*γ* may be associated with the cognition impairment. However, the expression and activity of PPAR-*γ* in central nervous system in diabetes still remains unclear. In the present study, we found that the constant hyperglycemia rats had a higher PPAR-*γ* expression in hippocampus and hypothalamus after 2 weeks of induction, whereas the constant hyperglycemia rats raised for 8 weeks after induction had a lower PPAR-*γ* expression in hippocampus and hypothalamus. We assume that glucose is receptor stimulant of the PPAR-*γ*. If the glucose is increasing, the PPAR-*γ* will be upregulated; however, if the glucose is too higher, the PPAR-*γ* will be down regulated. So PPAR-*γ* expression increased at 2 weeks post-STZ injection where it decreased after 8 weeks. The lower level of PPAR-*γ* in hippocampus and hypothalamus induced by chronic hyperglycemia might be related to the cognitive impairment in late diabetic patients. Furthermore, PPAR-*γ* agonist can inhibit the expression of 11*β*-HSD1 in 3T3-L1 adipocytes [18], and decrease 11*β*-HSD1 activity [19]. So we speculate that PPAR-*γ* agonist could reduce local GC levels in central nervous system caused by the decreased 11*β*-HSD1 levels or activity, which can contribute to the cognitive protection. Our study provides a new clue for the further study.

In our study we also observed that serum ACTH concentration in the constant hyperglycemia group was higher than that in the control group and the fluctuant glycemia group after 2 weeks of induction. And the serum ACTH concentration in the fluctuant glycemia group was also higher than that in the control group. As for the rats after 8 weeks of induction, the constant hyperglycemia group had a higher serum ACTH concentration and a lower serum corticosterone concentration, compared with that in the control group. The lower level of corticosterone might result from the injure of rat adrenal glands caused by the toxicity of STZ and hyperglycemia. ACTH was more sensitive to the stress of hyperglycemia than corticosterone feedback, so it had a higher concentration due to the hyperglycemia stress. It has been reported that chronically high level of glucocorticoids was deleterious and led to neuropsychiatric impairment, such as depression and psychosis [20]. However, our study showed that the hyperglycemia rats with cognitive impairment had lower serum corticosterone level. Taylor observed that, after treatment of carbenoxolone (CBX), a 11*β*-HSD1 inhibitor, for 16 days in mice, 11*β*-HSD1 gene expression was significantly downregulated in liver, but circulating plasma corticosterone levels were not altered by CBX [21]. Aged 11*β*-HSD1 knock-out mice had a better cognition compared to the wild type mice and had a higher circulating GC concentrations [15].

In summary, the cognition has a higher relationship with the local GC levels rather than the circulating GC levels.

## 5. Conclusions

Hyperglycemia increased 11*β*-HSD1 expression and decreased PPAR-*γ* expression in hippocampi and hypothalami after 8 weeks of induction, which probably correlated to the cognitive impairment of the constant hyperglycemia rats.

## Figures and Tables

**Table 1:** Serum concentrations of ACTH and corticosterone.

	ACTH (pg/mL)	Corticosterone (mmoL/L)
After 2 weeks of induction		
Control group (*n* = 10)	84.85 ± 12.50	17.18 ± 1.75
Constant hyperglycemia group (*n* = 10)	399.29 ± 101.40^∗∗^	20.95 ± 4.61
Fluctuant glycemia group (*n* = 10)	274.04 ± 80.40^∗∗##^	17.94 ± 6.76
After 8 weeks of induction		
Control group (*n* = 9)	44.88 (30.02)	47.89 ± 8.50
Constant hyperglycemia group (*n* = 9)	85.01 (38.90)^∗∗^	9.64 ± 1.05^∗∗^

All the data of ACTH or corticosterone after 2 weeks of induction were standardized into Z-scores and shown as the means ± SD, analyzed by one-way ANOVA. Serum ACTH level of the constant hyperglycemia group was higher than that in the control group (*P* < 0.01) and the fluctuant glycemia group (*P* < 0.01). Serum ACTH level in the fluctuant glycemia group was higher than that in the control group (*P* < 0.01). The data of ACTH after 8 weeks of induction were not standardized into Z-scores, shown as median (interquartile range) and analysed by the Wilcoxon rank sum test. The data of corticosterone were standardized into Z-scores and analyzed by one-way ANOVA. The constant hyperglycemia group had a lower serum corticosterone level (*P* < 0.01) and higher serum ACTH level, compared with the control group. ^∗∗^ *P* < 0.01, versus control group. ^##^ *P* < 0.01, versus constant hyperglycemia group.

## References

[1] Kamboj SS, Chopra K, Sandhir R (2008). Neuroprotective effect of N-acetylcysteine in the development of diabetic encephalopathy in streptozotocin-induced diabetes. *Metabolic Brain Disease*.

[2] Manschot SM, Biessels GJ, Cameron NE (2003). Angiotensin converting enzyme inhibition partially prevents deficits in water maze performance, hippocampal synaptic plasticity and cerebral blood flow in streptozotocin-diabetic rats. *Brain Research*.

[3] Nitta A, Murai R, Suzuki N (2002). Diabetic neuropathies in brain are induced by deficiency of BDNF. *Neurotoxicology and Teratology*.

[4] Munshi M, Grande L, Hayes M (2006). Cognitive dysfunction is associated with poor diabetes control in older adults. *Diabetes Care*.

[5] Pocai A, Lam TKT, Obici S (2006). Restoration of hypothalamic lipid sensing normalizes energy and glucose homeostasis in overfed rats. *Journal of Clinical Investigation*.

[6] Yau JLW, McNair KM, Noble J (2007). Enhanced hippocampal long-term potentiation and spatial learning in aged 11*β*-hydroxysteroid dehydrogenase type 1 knock-out mice. *Journal of Neuroscience*.

[7] Pelletier G, Luu-The V, Li S, Bujold G, Labrie F (2007). Localization and glucocorticoid regulation of 11*β*-hydroxysteroid dehydrogenase type 1 mRNA in the male mouse forebrain. *Neuroscience*.

[8] van Bilsen M, van der Vusse GJ, Gilde AJ, Lindhout M, van der Lee KAJM (2002). Peroxisome proliferator-activated receptors: lipid binding proteins controling gene expression. *Molecular and Cellular Biochemistry*.

[9] Vosper H, Khoudoli GA, Graham TL, Palmer CNA (2002). Peroxisome proliferator-activated receptor agonists, hyperlipidaemia, and atherosclerosis. *Pharmacology and Therapeutics*.

[10] Gao L, Chiou WJ, Camp HS, Burns DJ, Cheng X (2009). Quantitative measurements of corticosteroids in ex vivo samples using on-line SPE-LC/MS/MS. *Journal of Chromatography B*.

[11] Du J, Zhang L, Liu S (2009). PPAR*γ* transcriptionally regulates the expression of insulin-degrading enzyme in primary neurons. *Biochemical and Biophysical Research Communications*.

[12] Schaaf MJM, De Kloet ER, Vreugdenhil E (2000). Corticosterone effects on BDNF expression in the hippocampus implications for memory formation. *Stress*.

[13] Yau JLW, Noble J, Kenyon CJ (2001). Lack of tissue glucocorticoid reactivation in 11*β*-hydroxysteroid dehydrogenase type 1 knockout mice ameliorates age-related learning impairments. *Proceedings of the National Academy of Sciences of the United States of America*.

[14] Dumas TC, Gillette T, Ferguson D, Hamilton K, Sapolsky RM (2010). Anti-glucocorticoid gene therapy reverses the impairing effects of elevated corticosterone on spatial memory, hippocampal neuronal excitability, and synaptic plasticity. *Journal of Neuroscience*.

[15] Holmes MC, Seckl JR (2006). The role of 11*β*-hydroxysteroid dehydrogenases in the brain. *Molecular and Cellular Endocrinology*.

[16] Shimazu T, Inoue I, Araki N (2005). A peroxisome proliferator-activated receptor-*γ* agonist reduces infarct size in transient but not in permanent ischemia. *Stroke*.

[17] Zhang J, Ge H, Wang C (2007). Inhibitory effect of PPAR on the expression of EMMPRIN in macrophages and foam cells. *International Journal of Cardiology*.

[18] Berger J, Tanen M, Elbrecht A (2001). Peroxisome proliferator-activated receptor-*γ* ligands inhibit adipocyte 11*β*-hydroxysteroid dehydrogenase type 1 expression and activity. *Journal of Biological Chemistry*.

[19] Gurnell M (2007). “Striking the right balance” in targeting PPAR*γ* in the metabolic syndrome: novel insights from human genetic studies. *PPAR Research*.

[20] Seckl JR (2004). 11*β*-hydroxysteroid dehydrogenases: changing glucocorticoid action. *Current Opinion in Pharmacology*.

[21] Taylor A, Irwin N, McKillop AM, Flatt PR, Gault VA (2008). Sub-chronic administration of the 11*β*-HSD1 inhibitor, carbenoxolone, improves glucose tolerance and insulin sensitivity in mice with diet-induced obesity. *Biological Chemistry*.

